# The expanding role of SGLT2 inhibitors beyond glucose-lowering to cardiorenal protection

**DOI:** 10.1080/07853890.2020.1841281

**Published:** 2021-11-11

**Authors:** Emily Brown, John P. H. Wilding, Uazman Alam, Thomas M. Barber, Janaka Karalliedde, Daniel J. Cuthbertson

**Affiliations:** a Department of Metabolic and Cardiovascular Medicine, Institute of Life Course and Medical Sciences, University of Liverpool, Liverpool, UK; b Liverpool University Hospitals NHS Foundation Trust, Liverpool, UK; c Division of Diabetes, Endocrinology and Gastroenterology, Institute of Human Development, University of Manchester, Manchester, UK; d Human Metabolism Research Unit, University of Warwick, Coventry, UK; e University Hospital Coventry and Warwickshire NHS Trust, Coventry, UK; f School of Cardiovascular Medicine & Sciences, King’s College London, London, UK

## Abstract

The kidney plays a major physiological role in glucose homeostasis but also contributes to the pathophysiology of type 2 diabetes (T2D), mediated by renal sodium glucose cotransporters (SGLTs). This recognition led to the development of SGLT2 inhibitors that inhibit proximal renal tubular renal glucose and sodium reabsorption. The glucoretic and natriuretic effect of SGLT2 inhibitors is associated with reductions in HbA_1c_ levels, body weight, systolic blood pressure and triglycerides. Major vascular complications of T2D include cardiovascular disease and chronic kidney disease (CKD). Results from several cardiovascular outcome trials (CVOTs) with these drugs have highlighted benefits in reducing major adverse cardiovascular events by 11%, reducing the risk of cardiovascular death or hospitalization for heart failure (HF) by 23% and reducing the risk of progression of renal disease by 45%. Their cardiorenal benefits are apparent across a range of eGFRs (within CKD1-3 groups) and the presence or absence of ischaemic heart disease, HF or T2D. In patients with HF with reduced ejection fraction (HFrEF), similar risk reductions in cardiovascular death and HF events are also seen; results from studies in patients with HF with preserved ejection fraction (HFpEF) are awaited. Cardiorenal benefits have been recently reported in patients with CKD, regardless of the presence or absence of T2D. Indications for use of SGLT2 inhibitors have extended beyond glucose-lowering to a central role in cardiorenal protection. This review will first explore the mechanisms by which glycaemic control, weight loss and cardiovascular risk factors are modulated therapeutically with SGLT2 inhibitors. Subsequently, we outline putative mechanisms underpinning the cardiorenal benefits seen, including in HF and CKD, in the context of completed and ongoing clinical studies. Treatment strategies with SGLT2 inhibitors in individuals with CKD or HF, with and/or without T2D are increasingly appealing. Combination therapy with complementary therapeutic agents is also explored.

## Introduction

### The role of the kidney in glucose homeostasis and renal glucose transport

#### Glucose homeostasis

The various roles of the kidney include the excretion of metabolic waste products, maintenance of intravascular volume and serum osmolality, regulation of blood pressure, acid-base and electrolyte balance through filtration, secretion and selective reabsorption of key ions. The kidney also has a major physiological role in regulating glucose homeostasis constantly supplying glucose to the brain despite diurnal changes in nutrient availability. The involvement of the kidney in gluconeogenesis was first described in 1938 in rabbits, where the amount of glucose required to maintain euglycemia was considerably higher after hepatectomy plus nephrectomy versus hepatectomy only [1].

Maintenance of whole-body glucose homeostasis involves several complementary inter-organ physiological processes, including glucose absorption (gastrointestinal tract), glycogenolysis (liver), gluconeogenesis (liver and kidneys), glucose reabsorption and excretion (kidneys). Renal glucose utilization, occurring predominantly in the renal medulla, constitutes ∼10% of whole-body glucose uptake to satisfy the kidneys’ energy requirements, while renal glucose release into the systemic circulation, via glycogenolysis and gluconeogenesis, is limited to the renal cortex. The kidney contributes up to 20% of glucose released into the systemic circulation under post-absorptive conditions, increasing up to 60% post-prandially [2]. These distinct metabolic roles reflect differences in the anatomical distribution of various enzymes along the nephron. Evidence suggests that in patients with T2D, renal gluconeogenesis is increased (versus healthy controls) in both the post-prandial and post-absorptive states [3].

#### Glucose filtration and reabsorption

**Figure 1.**
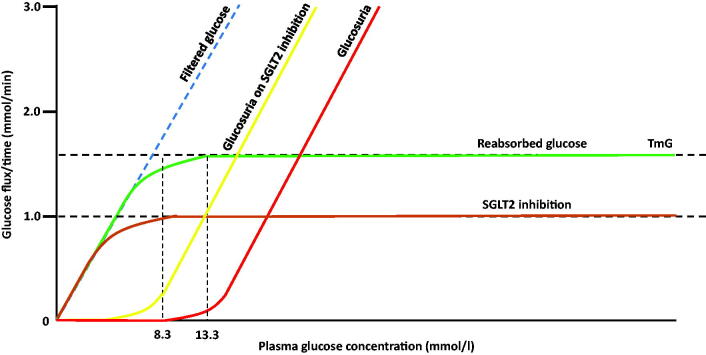
Renal glucose handling.

The kidneys also contribute to glucose homeostasis by filtering and reabsorbing glucose. The total amount of glucose stored in the body is ∼450 g, with ∼170 g of glucose produced daily (via gluconeogenesis and glycogenolysis). With glomerular filtration of 180 l/day, the kidney filters 162 g of glucose (180 l × 90 mg/dl; 90 mg/dl∼5.0 mmol/l) daily. The amount of glucose filtered increases linearly with increasing plasma glucose concentration; glucose is almost entirely reabsorbed in the proximal renal tubules. The renal threshold for reabsorption is a plasma glucose concentration ∼8.3 mmol/l, above which glucose starts to appear in the urine. Once plasma glucose concentration is >13.3 mmol/l, the maximum capacity for glucose reabsorption is exceeded (tubular maximum for glucose, TmG), and thus beyond this threshold, the degree of glucosuria increases in a linear fashion with increasing plasma glucose concentrations (Figure 1).

#### Glucose transport

**Figure 2.**
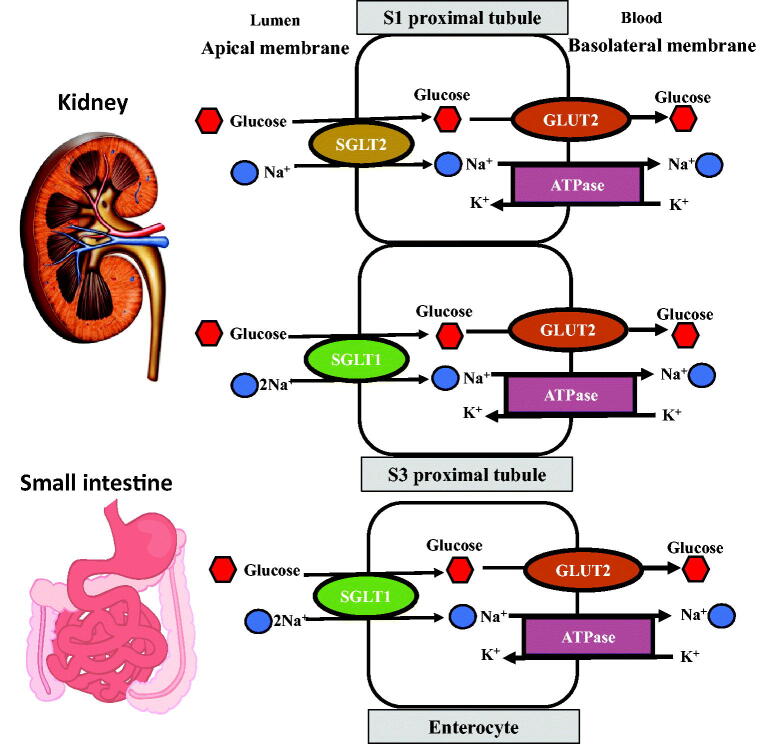
Glucose transport mechanisms in segments S1 and S2 of the renal tubules and in the enterocyte of the small intestine.

Glucose transport across cell membranes is dependent on two specialized carrier protein families: the facilitated glucose transporters (GLUTs) and the sodium glucose cotransporters (SGLTs). GLUTs are responsible for passive transport across cell membranes to equilibrate the transmembrane concentration while SGLTs are involved in active glucose transport across a concentration gradient. There are two predominant isoforms of SGLTs: SGLT1 and SGLT2. SGLT1 is located in the small intestine (in the luminal brush border of the enterocyte) and in segment 3 of the proximal tubule, with specificity for glucose and galactose as a high affinity (*K_m_* = 0.4 mM), low capacity glucose carrier. SGLT2 is found in the renal proximal tubules (segments 1 and 2), with specificity for glucose as a low affinity (*K_m_* = 2mM), high capacity glucose carrier. Thus, under normoglycaemic conditions, SGLT2, expressed in the early proximal tubule, accounts for ∼90% of glucose reabsorption with the remainder by SGLT1 in segment 3 of the proximal tubule (Figure 2).

#### Renal glucose transport in T2D

Evidence from studies conducted in human renal tubular cells suggests SGLT2 and GLUT2 expression are upregulated in T2D, leading to an increased maximal capacity for glucose reabsorption (TmG) and a higher threshold for glycosuria and consequently increased glucose reabsorption [4]. Increased renal glucose reabsorption is a well-recognized pathophysiological defect of T2D [5].

### SGLT2 inhibitors

#### SGLT1/2 receptor specificity

The appreciation of the role of the kidney in regulating glucose homeostasis led to the development of new types of glucose-lowering drugs targeting this metabolic pathway. The original observations that augmenting renal glucose excretion could lead to improved glycaemic control came from animal studies using phlorizin, a naturally occurring molecule, that inhibits both SGLT1 and SGLT2 and subsequently several SGLT2 and later dual SGLT1/2 inhibitors were developed. SGLT2 inhibitors in clinical use in Europe are dapagliflozin, canagliflozin, empagliflozin and ertugliflozin while sotagliflozin, the only dual SGLT1/2 inhibitor, is approved only for type 1 diabetes (T1D).

The differing SGLT2 inhibitors have a range of relative specificities to the different SGLT receptors which may contribute to subtle differences in their clinical profiles. The highest selectivity for SGLT2 receptors is observed with empagliflozin (SGLT2:SGLT1 specificity∼2500), with other agents intermediate in SGLT2 receptor specificity (dapagliflozin,1200; canagliflozin, 200) with sotagliflozin the least selective (∼20).

Despite our understanding that SGLT2 usually contributes to ∼90% of glucose reabsorption in a healthy adult, pharmacological inhibition with an SGLT2 inhibitor only results in about 60–80 g of urinary glucose excretion (∼50 to 60% of filtered glucose load). With increased glucose load to the late proximal tubule, SGLT2 inhibition causes a compensatory increase in SGLT1-mediated glucose reabsorption to limit glucosuria and potential hypoglycaemia [6]. Additionally, inhibition of intestinal SGLT1-mediated glucose uptake may have further therapeutic benefits. This premise is supported by findings from studies in individuals who carry a haplotype of functionally damaging missense mutations in SGLT1, which due to reduced intestinal glucose uptake, have improved glucose tolerance and lower odds of impaired glucose tolerance [7]. The estimated 25-year effect of this reduction in glucose tolerance was concomitant reductions in prevalent obesity, incident diabetes, heart failure (HF) and death [7].

#### Metabolic effects

SGLT2 inhibitors were introduced as a treatment for T2D but by virtue of the associated urinary excretion of glucose (and thus caloric loss), and urinary excretion of sodium, their use is associated with reductions in HbA_1c_ levels, body weight and systolic blood pressure.

#### Glycaemic effects

All SGLT2 inhibitors show similar reductions in HbA_1c_ when studied as monotherapy in drug-naive patients [8,9], in combination with other oral agents [10–13] or insulin [14]. Although the short-term reduction in HbA_1c_ with SGLT2 inhibitors is comparable to that achieved with sulphonylureas and DPP-IV inhibitors (0.7–1%), the durability of the glycaemic benefit appears to be better with SGLT2 inhibitors compared to these other drug classes [15,16]

#### Factors predicting glycaemic response

A meta-analysis of randomized controlled studies, confirms that the magnitude of HbA_1c_ reduction correlates with the baseline HbA_1c_, irrespective of the class or mode of action of glucose-lowering therapy, such that patients with higher HbA_1c_ achieve greater reductions than individuals with lower HbA_1c_ [17]. However, this finding is particularly pertinent considering the efficacy of SGLT2 inhibitors, where with progressively higher baseline HbA_1c_, their glucose-lowering effect not only increases but indeed exceeds that of other glucose-lowering therapies. Mechanistically, this is explained by significantly higher amounts of glucose filtered whose reabsorption can then be targeted by SGLT2 inhibitor therapy as the plasma glucose concentration and HbA_1c_ progressively increases. Considering that their primary effect is mediated by inhibiting renal glucose absorption, provided that renal function (and thus glomerular filtration) is relatively preserved, these agents effect similar improvements in glycaemic control in newly diagnosed or longer duration T2D, irrespective of their degree of insulin sensitivity or residual insulin secretion [18]. Renal impairment, (and thus impairment in glucose filtration), is associated with reduced efficacy of SGLT2 inhibitors in glycaemic reduction.

#### Mechanisms of glucose-lowering

There are two mechanisms by which SGLT2 inhibitors improve glycaemic control. First, by their significant effects on modulating renal glucose excretion: reducing the maximal capacity for glucose reabsorption (TmG) and the threshold for glycosuria, promoting glycosuria of 60–80 g/day [19]. Secondly, by amelioration of glucotoxicity (achieved through the reduction in plasma glucose concentration secondary to glycosuria) leading to greater insulin sensitivity in peripheral tissues (adipose tissue and skeletal muscle) [20] and enhancement of beta-cell function, with improvements in the first and second phase of insulin secretion [21,22]. These favourable metabolic changes are partially offset by an increase in endogenous glucose production, possibly the result of an increase in plasma glucagon concentrations. As their mechanism of action is independent of insulin secretion their use is associated with a low incidence of hypoglycaemia and therefore, they may be added to any background glucose-lowering treatment regimen. They may cause hypoglycaemia when used in combination with a sulphonylurea or insulin therapy.

#### Weight loss

The weight loss associated with SGLT inhibitors is typically around 2–3 kg after 6 months’ treatment, the magnitude of which has been the subject of several meta-analyses [23]. Arguably, the weight loss associated with 300 mg canagliflozin is marginally greater than that of most other SGLT2 inhibitors [24]. The changes in body composition associated with this weight loss have been quantified and there is a reduction in total fat mass, visceral and subcutaneous adipose tissue [25]. The anticipated weight loss from SGLT2 inhibitor therapy has been calculated based on the known urinary glucose excretion of ∼60 to 80 g per day, which amounts to a caloric loss of 240–320 calories per day (glucose∼4 kcal/g). Considering 3500 calories is equivalent to 1 lb of fat (0.45 kg) the observed magnitude of weight loss is significantly less than anticipated if one looks at the longer-term weight loss (e.g. after 6 months). Based on results of studies in animals and humans treated with SGLT2 inhibitors, it has been suggested that compensatory hyperphagia explains the attenuation of weight loss [26] and Hall et al. have modelled this to determine that every kilogram of weight loss results in a proportional increase in appetite resulting in eating above baseline by approximately 100 kcal/day [27]. This concept of compensatory hyperphagia with SGLT2 inhibitor therapy provides the rationale for the co-administration of an SGLT2 inhibitor with a GLP-1 receptor agonist to attenuate the central hyperphagic drive and ideally achieve additive, even synergistic, weight loss. Mechanistic explanations for weight loss associated with SGLT2 inhibitors, with or without GLP1 receptor agonists have been discussed narratively [28].

#### Mechanisms of weight loss

The mechanisms for weight loss are predominantly through urinary glucose (and associated caloric loss), natriuresis and aquaresis but there are other additional mechanisms. Reproducible changes in substrate utilization such that fuel use switches from glucose oxidation to increased lipolysis, fat oxidation and formation of ketone bodies. Furthermore, in patients treated with SGLT2 inhibitors and sulphonylureas and/or insulin, the reduction in their dosage may also contribute to favourable weight change.

#### Blood pressure reduction

There are consistent sustained reductions in systolic (∼5mmHg) and diastolic (∼2mmHg) blood pressure with SGLT2 inhibitors, similar across all members of the class. With the coupling of glucose and sodium reabsorption in the proximal tubule, SGLT2 inhibition leads to osmotic diuresis and mild natriuresis and a corresponding contraction in extracellular fluid and plasma volume [29]. These blood pressure lowering effects may extend to individuals without T2D [30].

Almost certainly it is a multi-factorial combination of mechanisms that contribute to the pathophysiology of CVD and CKD in T2D including the metabolic and haemodynamic abnormalities described above, together with other mechanisms such as lipotoxicity and oxidative stress; it is likely that their improvement/reversal explains the clinical benefits of SGLT2 inhibitors.

### Effects of SGLT2 inhibitors on the cardiovascular system

T2D is a major cardiovascular risk factor, conferring a two-to three-fold excess risk of coronary artery disease including angina, myocardial infarction, stroke and HF, in patients with and without the established cardiovascular disease [31,32]. There has been much focus on understanding atherosclerotic complications (atherosclerotic CVD, ASCVD), with the relationship between T2D and HF less well understood.

#### Effects on cardiovascular outcomes

These initial studies described below, mandated by the FDA, aimed to demonstrate that SGLT2 inhibitors could be used in patients with T2D without causing an increase in major adverse cardiovascular events (MACE). The subsequent results of several of these key CVOTs have consistently reassured the medical community about the safety of SGLT2 inhibitors and the associated reduction in cardiovascular events in patients with established CVD or at high risk of CV events. The discovery of their beneficial role in HF was somewhat serendipitous [33]. Recent guidelines, updated to reflect these overwhelmingly positive results, recommend these drugs in patients either with T2D and CVD *or* at very high/high CV risk to reduce CV events and to reduce the risk of death [34].

#### EMPA-REG cardiovascular outcome study

The EMPA-REG cardiovascular outcome trial (CVOT) assessed the effect of empagliflozin (10 mg or 25 mg once daily), compared with placebo, both added to standard of care comprising glucose-lowering agents and cardiovascular (CV) drugs (including antihypertensive and lipid-lowering agents) in 7020 patients over a median of 3.1 years. The clinical population was a high-risk clinical group with almost all having pre-existing CVD (∼99%). The composite primary endpoint was a 3-point MACE (defined as time to the first occurrence of CV death, non-fatal heart attack, or non-fatal stroke). Empagliflozin significantly reduced the occurrence of the 3-point MACE by 14% versus placebo. The risk of CV and all-cause mortality was reduced by 38 and 32%, respectively, with no significant difference in the risk of non-fatal heart attack or non-fatal stroke. The benefits were observed with almost immediate effect following the initiation of empagliflozin treatment [35]. Subsequent trials with other SGLT2 inhibitors confirmed these observations across a broader range of patients with and without established vascular disease.

#### CANVAS cardiovascular outcome study

The Canagliflozin Cardiovascular Assessment Study (CANVAS) CVOT assessed the effect of canagliflozin (100 mg or 300 mg), compared with placebo, both added to standard of care in 10,142 patients with T2D and high cardiovascular risk (65.6% had a history of cardiovascular disease) [36]. Patients were followed for a mean of 188.2 weeks. The composite primary endpoint was a 3-point MACE and canagliflozin significantly reduced the risk of the composite measure by 14% versus placebo. However, as in EMPA-REG the individual effects on CV death, non-fatal MI, or non-fatal stroke did not reach statistical significance. Canagliflozin also caused a 33% reduction for HF. Adverse reactions included an increased risk of amputations, primarily at the level of the toe or metatarsal.

#### DECLARE-TIMI

The Dapagliflozin to assess Cardiovascular Events trial was the largest study to assess the effect of an SGLT2 inhibitor, namely dapagliflozin, in 17,160 patients with established CVD (40.6%) or with multiple risk factors for atherosclerotic CVD (59.4%) (i.e. primary and secondary prevention) for a median of 4.2 years [37]. Dapagliflozin demonstrated non-inferiority for MACE (hazard ratio [HR] 0.93; 95% CI 0.84, 1.03) but resulted in a lower rate of a composite of CV death or hospitalization for HF (HR 0.83; 95% CI 0.73, 0.95), due to a lower rate hospitalization for HF, regardless of previous ASCVD or HF (HR 0.73; 95% CI 0.61, 0.88). These studies have led to the approval of SGLT2 inhibitors for the indication of a reduction in risk of CV events and death in patients with T2D.

#### Meta-analyses of SGLT2 CVOTs

**Figure 3.**
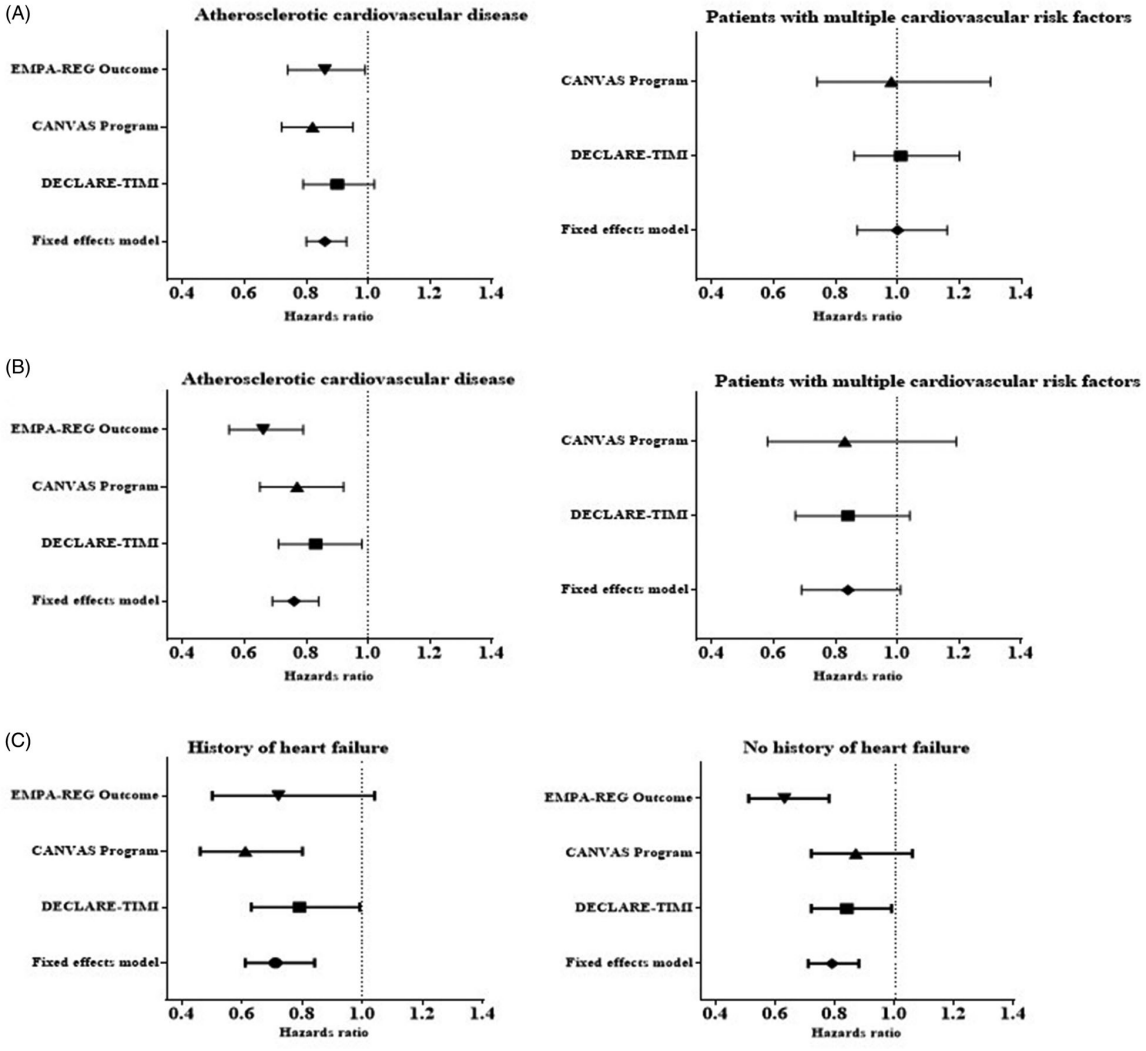
(A) Effect on MACE stratified by the presence of established atherosclerotic CVD or multiple risk factors, (B) Effect on hospitalization for HF and CV death stratified according to history of atherosclerotic CVD or multiple risk factors, (C) Effect on hospitalization for HF and CV death stratified according to history of HF [39].

Several meta-analyses have recently been published on SGLT2 inhibitors for primary and secondary prevention of cardiovascular outcomes in T2D with an attempt to reconcile what patient characteristics (e.g. presence/absence of atherosclerotic cardiovascular disease (ASCVD), history of HF or baseline renal function) will derive cardiorenal protection [38,39]. Incorporating data from 34,322 patients in EMPA-REG OUTCOME, CANVAS and DECLARE-TIMI 58, the clinical benefits of SGLT2 inhibitors are in reducing the risk of a 3-point MACE (MI, stroke or cardiovascular death) only in those with established CVD (HR 0.86; 95% CI 0.80, 0.93) and not in those with multiple risk factors (HR 1.00; 95% CI 0.87, 1.16) (Figure 3).

Cardiovascular safety of the dual SGLT1/SGLT2 inhibitor sotagliflozin is being tested in the SCORED trial in patients with T2D, moderate renal impairment and high cardiovascular risk (NCT 03315143).

##### Effects on HF

HF is a major public health issue affecting up to 63 million people worldwide [40], with 1 in 5 people expected to develop HF during their lifetime [41]. T2D is a common co-morbidity in HF patients: not only is T2D a major risk factor for the development of HF but it also represents a major adverse prognostic factor in those with established HF [42,43]. Chronic HF is the leading cause of hospitalization in patients over 65 years old [44], with those hospitalized having a 10%, 30-day and 50%, 1-year mortality.

Patients with T2D may develop two distinct phenotypes of HF according to their ejection fraction.

##### HFrEF

Many develop HF with a reduced ejection fraction (EF < 40%, HFrEF), typically characterized by a loss and stretch of cardiac myocytes, left ventricular enlargement and increased serum natriuretic peptides; these patients respond to treatment with neurohormonal antagonists (inhibitors of the renin-angiotensin system including ACE-inhibitors, angiotensin receptor blockers, angiotensin receptor neprilysin inhibitors, beta-blockers and mineralocorticoid receptor antagonists, MRA).

##### HFpEF

Others will develop HF with preserved ejection fraction (EF > 50%; HFpEF) characterized by systemic and adipose tissue inflammation, microvascular dysfunction and myocardial fibrosis. In contrast, such patients do not have significantly increased LV size or concentrations of serum natriuretic peptides and show little/no response to neurohormonal antagonists [45]. There is an additional intermediate phenotype with midrange EF 40-50%. Observational studies highlight a shifting pattern of the epidemic with the prevalence of HFpEF increasing relative to HFrEF [46], likely to constitute ∼65% of the total HF burden. This is consistent with the increasing prevalence of comorbidities such as T2D, obesity, hypertension, etc. that drive disease progression in HFpEF. Considering this rising prevalence there is a need for recognition of individuals at risk and identifying the presence of preclinical cardiac structural/functional HF may help to prevent progression and improve clinical outcomes.

#### Effects of glucose-lowering therapies

Metformin has been associated with positive effects in HF, certain DPP-4 inhibitors (saxagliptin) and thiazolidinediones are not recommended and the evidence for GLP-1 agonists is inconclusive while results from four large-scale clinical trials of >36,000 patients with T2D suggest that SGLT2 inhibitors have a role in the prevention of HF [39]. The three main SGLT2 inhibitors (empagliflozin, canagliflozin and dapagliflozin) lowered the risk of hospitalization for HF by ∼25 to 35%, with the benefits almost immediately apparent on the initiation of treatment and persisted throughout the follow-up period of 2–5 years. Only 10–15% of these trial patients had documented HF around the time of randomization. Significantly a similar benefit was observed in patients with and without atherosclerotic cardiovascular disease and with and without a history of HF [39]. From meta-analyses of SGLT2 inhibitors and HF from the various CVOTs, the risk reduction in hospitalization for HF was evident regardless of the presence of ASCVD or HF at baseline [39] (Figure 3). Significantly, analysis of data from DECLARE and CANVAS suggested the risk of hospitalization for HF was reduced in patients with and without HFrEF suggesting potential benefit in patients with HFpEF in whom no treatment has been shown to be of benefit [47,48].

#### DAPA-HF

Recently a large randomized, controlled study (DAPA-HF) of dapagliflozin 10 mg once daily was undertaken in 4744 patients, mean age 66 years, with symptomatic HF and reduced ejection fraction (HFrEF); mean baseline EF was ∼31%. The patients were already receiving excellent background HF therapy with neurohormonal antagonists (>90% renin-angiotensin system inhibitors, >70% MRAs). Over a median follow-up of 18.2 months dapagliflozin reduced the risk of the primary composite end-point of cardiovascular death and HF events (hospitalization or an ambulatory visit for intravenous diuretic therapy) by 26% [49]. CV death was significantly reduced by 18% and all-cause mortality by 17%. Significantly only 42% had T2D and >40% did not have underlying ischaemic heart disease. The magnitude of clinical benefit on the primary outcome was observed irrespective of age and was similar in patients with or without T2D and with or without ischaemic heart disease. The number needed to treat (over 18 months) was only 21, supporting a large absolute risk reduction.

### EMPA-RESPONSE-AHF

To test the hypothesis that SGLT2 inhibitors may be effective in patients with acute decompensated HF, a pilot study with empagliflozin was performed. In this randomized, placebo-controlled, double-blind, parallel-group, multicentre study, 80 acute HF patients, with and without diabetes, were randomized to receive either empagliflozin 10 mg/day or placebo for 30 days. Although empagliflozin had no effect on change in VAS dyspnoea, diuretic response, serum NT-proBNP and length of hospital stay it was deemed to be safe, increased urinary output and reduced a combined endpoint of in-hospital worsening HF, rehospitalization for HF or death at 60 days compared with placebo [4 (10%) versus 13 (33%); *p* = .014] [50].

The implications of the findings of these studies are that SGLT2 inhibitors appear to reduce the risk of hospitalization for HF in patients both without a pre-existing diagnosis of HF and in those with established HF; applicable for non-diabetic patients and those with T2D. In May 2020, dapagliflozin was the first SGLT2 inhibitor to be approved for the use of HF with reduced ejection fraction by the Federal Drug Administration (FDA).

#### Current HF studies

**Figure 4.**
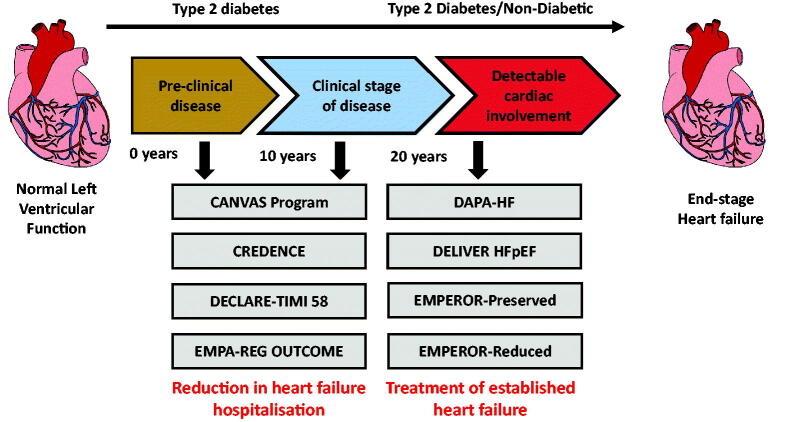
Summary of the main cardiovascular outcome trials.

There are several ongoing studies of SGLT2 inhibition in patient cohorts with different phenotypes of HF (Figure 4). The EMPEROR-Reduced trial is examining the effect of empagliflozin in patients, with and without T2D, with more advanced HF (lower LV ejection fraction and higher serum concentrations of natriuretic peptides). EMPEROR-Preserved with empagliflozin and DELIVER with dapagliflozin will evaluate the effects of SGLT2 inhibition in patients with an established diagnosis of HFpEF. If these studies highlight a beneficial impact on HF events in HFpEF this will substantially broaden the clinical indications of these drugs.

#### Effects on atrial fibrillation

In a recent analysis of DECLARE, dapagliflozin was shown to decrease the incidence of reported episodes of atrial fibrillation/atrial flutter (AF/AFL) adverse events in high-risk patients with T2D. This effect was consistent regardless of the patients’ prior history of AF, ASCVD, or HF [51].

##### Observational data of cardiovascular effects of SGLT2 inhibitors

Several observational, population-based, retrospective cohort studies have examined the effects of SGLT2 inhibitors on rates of death and/or hospitalization for HF in patients with T2D [52,53]. In the THIN database from the UK, patients with T2D who were exposed to dapagliflozin had a lower risk of death from any cause (i.e. lower all-cause mortality) irrespective of baseline CVD status [53]. Similarly, in the CVD-REAL study, data were collected from medical claims, primary care/hospital records and national registries from the US, Norway, Denmark, Sweden, Germany and the UK. Treatment with any SGLT2 inhibitor (canagliflozin, dapagliflozin and empagliflozin) versus other oral glucose-lowering drug treatments was associated with lower rates of hospitalization for HF and death [52]. These findings have now been replicated in a further study from the Nordic registers [54]. A subsequent study, the CVD REAL 2 study, found that use of SGLT2 inhibitors (versus other glucose-lowering drugs) in patients with T2D from Asia, the Middle East and North America was associated with a lower risk of all-cause death, hospitalization for HF, MI and stroke [55].

##### Potential mechanisms of cardiovascular benefit of SGLT2 inhibitors

The mechanism by SGLT2 inhibitors mediates this reduction in CV death and HF remains unknown, although many theories have been proposed [56]. The findings, both in their nature and speed of onset, clearly implicate non-glycaemic mechanisms of benefit. Although T2D is a major risk factor for CVD, there is no evidence from this study, or indeed from any CV outcome study that the glucose-lowering action mediates the beneficial effect in reducing CVD, HF or deaths. This is particularly reinforced by the findings of DAPA-HF where similar benefits were observed in those with or without T2D. For similar reasons, the effects do not appear to be mediated by *anti-atherogenic effects*. Proposed cardiac mechanisms include cardiac remodelling, improved contractility and a shift in myocardial and renal substrate utilization from fat and glucose oxidation towards an energy-efficient “super fuel” like ketone bodies, which improve myocardial/renal work efficiency and function [57,58]. Preservation of renal function may also help but a diuretic or natriuretic effect appears unlikely as the intensification of diuretic therapy in HF is associated with an increased risk of CV and sudden death and in DAPA-HF, dapagliflozin, only produced a 10–15% decline in NT-proBNP levels. Sustained volume contraction, by reducing myocardial stretch, may attenuate susceptibility to arrhythmias.

The collective CV data and clinical trials context are summarised in Figures 3 and 4.

##### Effects of SGLT2 inhibitors on chronic kidney disease

CKD, as defined by the presence of kidney damage or glomerular filtration rate (GFR < 60 ml/min/1.73 m^2^) for 3 months, is classified into stages based on the level of GFR [59]. CKD affects 700 million individuals worldwide and contributes to 1 in 20 deaths annually [60]. As well as HF, T2D is frequently complicated by CKD (∼40% of people with T2D) and T2D is now the leading cause of end-stage kidney disease (ESKD) globally. Indeed, these three entities (T2D, HF and CKD) are interconnected frequently co-existing such that 50% of individuals with HF have moderate-severe CKD. The co-existence of CKD and HF reflects many common aetiologies including advancing age, hypertension, coronary artery disease, and T2D [61]. The presence of any one of these conditions worsens the other two conditions [62]; hospitalizations due to HF are associated with *a* > 11-fold increased risk of end-stage renal disease compared with patients who did not have CVD [63]. Furthermore, the presence of CKD in HF is associated with increased morbidity and mortality with a higher risk of all-cause death, CV death and hospitalization for HF [64]. Significantly, the risk of death in HF is more strongly associated with a decline in eGFR than in ejection fraction [65].

**Figure 5.**
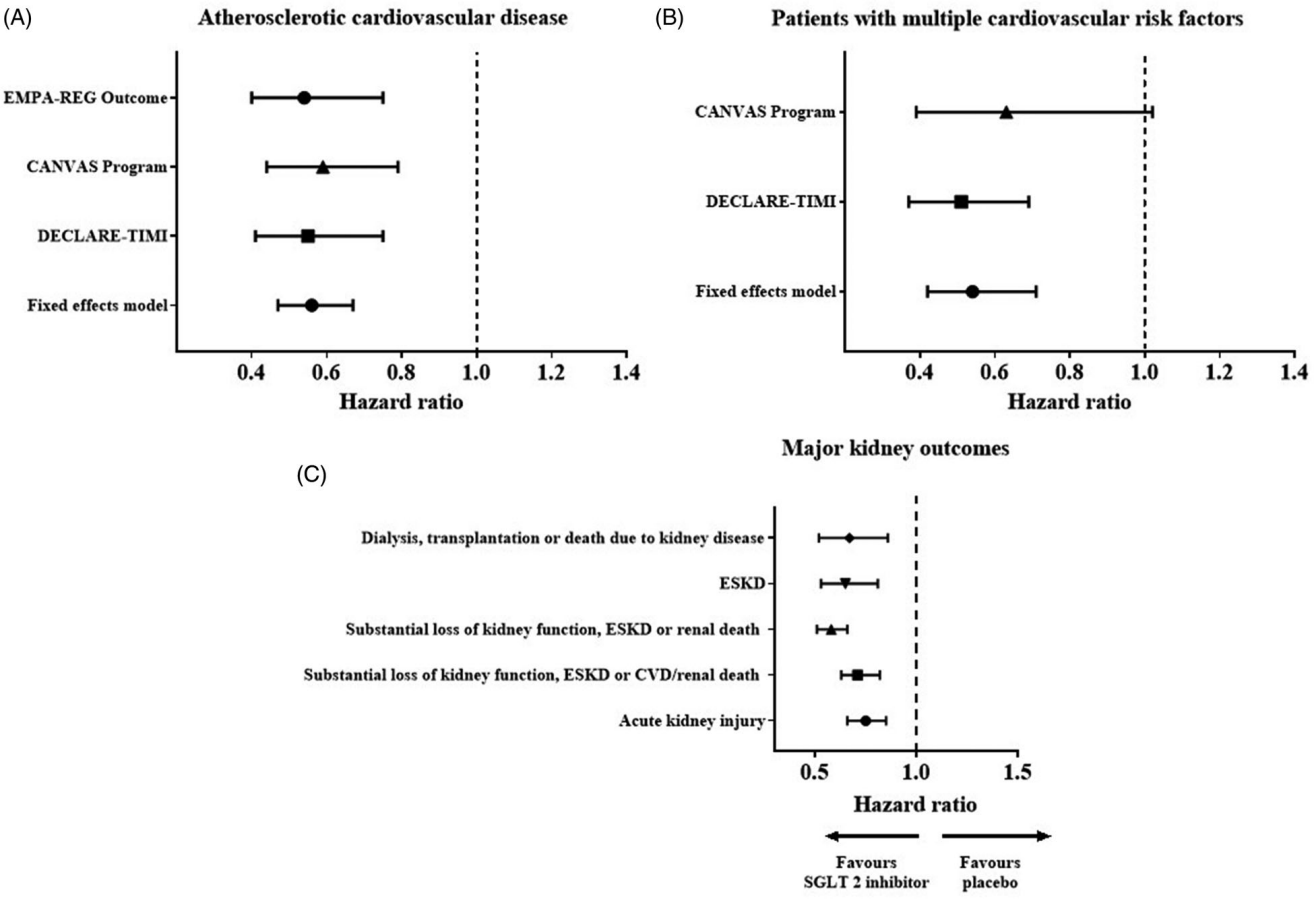
Summary of the effects of the SGLT2 inhibitors on the major kidney outcomes (A) and (B) from EMPA-REG, CANVAS and DECLARE, (C) also incorporating CREDENCE data [39,68].

In recent decades despite attempts to achieve optimal glycaemic and blood pressure control, and widespread use of ACEs/ARBs, there remains a high residual risk for patients with CKD to progress to end-stage kidney disease (ESKD) which highlights the need for additional renoprotective therapies to preserve eGFR and prevent ESKD. The renoprotective effects of SGLT2 inhibitors were first demonstrated in EMPA-REG, DECLARE and CANVAS [35,37,66] although all renal outcomes reported were secondary outcome measures. In a meta-analysis of these three CVOTs, SGLT2 inhibitors reduced the composite of worsening renal function, end-stage renal disease or renal death by 45% (HR 0.55; 95% CI 0.48, 0.64) with a similar effect whether studied in patients with atherosclerotic CVD or those with multiple risk factors [39] (Figure 5). Renoprotection was evident across all baseline GFR levels but with a progressively attenuated effect as eGFR declined. Importantly, these studies enrolled only a small fraction of patients with CKD at baseline (the lowest mean baseline eGFR was 74 ml/min/1.73 m^2^ in EMPA-REG, 76.5 ml/min/1.73 m^2^ in CANVAS and 85 ml/min/1.73 m^2^ in DECLARE). Furthermore, fewer than 1/6 of participants have eGFR <60 ml/min/1.73 m^2^ with only a small proportion with baseline eGFR <45 ml/min/1.73 m^2^. Recognizing the need to study the participants with the highest risk of adverse renal outcomes (i.e. those with the lowest eGFR), and thus the group who would derive the most benefit, studies of SGLT2 inhibitors have been conducted or are underway. Currently, SGLT2 inhibitors are not approved for use in patients with eGFR < 45 ml/min/1.73 m^2^ based on the diminished glycaemic efficacy with impaired renal function.

The only currently published RCT designed to evaluate the renoprotective effects of an SGLT2 inhibitor in 4401 people with T2D, CKD, and macroalbuminuria, compared with a placebo, is the CREDENCE study (Evaluation of the Effects of Canagliflozin on Renal and Cardiovascular Outcomes in participants with Diabetic Nephropathy) [67]. This study was designed specifically to examine the effects of SGLT2 inhibition on renal outcomes in patients with T2D at high risk of renal disease progression. Inclusion criteria were eGFR > 30 and <90 ml/min/1.73 m^2^ with UACR >300–5000 mg/g with all patients receiving renin-angiotensin system blockade with stable doses of ACEs/ARBs. Mean eGFR was 56.2 ml/min/1.73 m^2^ with 60% had an eGFR of 30–60 ml/min/1.73 m^2^. The primary outcome measure was a composite of ESKD (dialysis, transplantation or a sustained eGFR of <15 ml/min/1.73 m^2^), a doubling of serum creatinine or death from renal or cardiovascular causes. Secondary outcomes included cardiovascular outcomes. This study was terminated early after reaching the pre-specified efficacy criteria. The relative risk of the primary outcome was reduced by 30% in the canagliflozin group relative to placebo with a 20–30% lower relative risk of deleterious cardiovascular outcomes. It also provided reassurance about the risk of amputation/fracture with similar rates between the two groups. This study demonstrated benefit for patients of all eGFR groups including those with a low baseline eGFR (eGFR 30–45 ml/min/1.73 m^2^ in whom SGLT2 inhibitors would not currently be recommended).

A further systematic review and meta-analysis which pooled data from the 3 CVOTs and CREDENCE, including a total of 38,723 participants, demonstrated consistent reductions in all the major kidney outcomes including acute kidney injury [68] (Figure 5). Significantly, there was clear separate evidence of benefit across all groups, irrespective of baseline eGFR, albuminuria or use of RAS blockade. The evidence from CREDENCE and this meta-analysis highlight a renoprotective role for patients with T2D and eGFR of 30–45 ml/min/1.73 m^2^.

##### Studies of SGLT2 inhibition and CKD without T2D

The results of the DAPA-CKD trial have extended these findings to the broader population of patients with CKD (including participants with an eGFR 25–75 ml/min/1.73 m^2^), consisting approximately two-thirds of the population with and one third without T2D, and demonstrated that the SGLT2 inhibitor, dapagliflozin, conferred kidney protection in patients with CKD. The occurrence of the composite primary outcome measure of a sustained decline in eGFR of at least 50%, end-stage kidney disease or death from renal/cardiovascular causes was reduced by 44%. Similarly, the risk of a composite of death from cardiovascular causes or hospitalization for heart failure was reduced by 29%.[69].

A short-term (6 week) randomized double-blind, placebo-controlled, cross-over study (the DIAMOND study) has examined the effects of dapagliflozin on proteinuria in non-diabetic patients with CKD and on stable RAS blockade. Although this did not show a difference in mean proteinuria change from baseline between dapagliflozin and placebo, this may reflect the short study duration and the outcomes of longer-term clinical trials are awaited [70].

##### Current renal outcome studies

EMPA-KIDNEY evaluating empagliflozin in patients with CKD, with or without T2D, is currently underway. Results from this study, with the results from DAPA-CKD, will likely pave the way for their application in patients with CKD, irrespective of a background of T2D.

##### Observational studies

In clinical practice, there is a more heterogeneous population of patients that receive SGLT2 inhibitors than those randomized in placebo-controlled clinical trials and thus real-life studies assess whether clinical trial data extends to patients treated in routine clinical practice. CVD-REAL 3 was a multi-national, observational cohort study examining new users of SGLT2 inhibitors with other glucose-lowering drugs with measurements of eGFR before and 180 days after initiation [71]. During follow up, SGLT2 inhibitor initiation was associated with reduced eGFR decline and a lower risk of major kidney events compared with initiation of other glucose-lowering drugs.

In another such study, using nationwide data from routine clinical practice in Scandinavia (Sweden, Denmark and Norway) Pasternak et al., demonstrated that initiation of SGLT2 inhibitors, compared with dipeptidyl peptidase-4 inhibitors using propensity matching, was associated with a significantly reduced risk of renal events reflecting a composite including renal replacement therapy, death from renal causes and hospital admission for renal events (3.6 fewer events per 1000 patient-years; HR 0.42, 95% CI 0.34, 0.53) [72]. These results suggest results in routine clinical practice are broadly similar to those observed in clinical trials.

##### Potential mechanisms of renal benefits of SGLT2 inhibitors

Our understanding of the underlying mechanisms through which SGLT2 inhibitors achieve renal protection is evolving [73]. Good glycaemic control is critical to preventing diabetic kidney disease and so metabolic effects with glucose-lowering, improved insulin sensitivity, and lesser glucose toxicity all contribute beneficially but there are multiple important non-glycaemic mechanisms of action. Some of these reflect direct effects of SGLT2 inhibition on the kidney mediated by the natriuretic effects including the normalisation of glomerular haemodynamics through the restoration of tubuloglomerular feedback (TGF), anti-inflammatory and anti-fibrotic actions and improving renal energy efficiency. Others relate to indirect effects including reductions in blood pressure and body weight.

**Figure 6.**
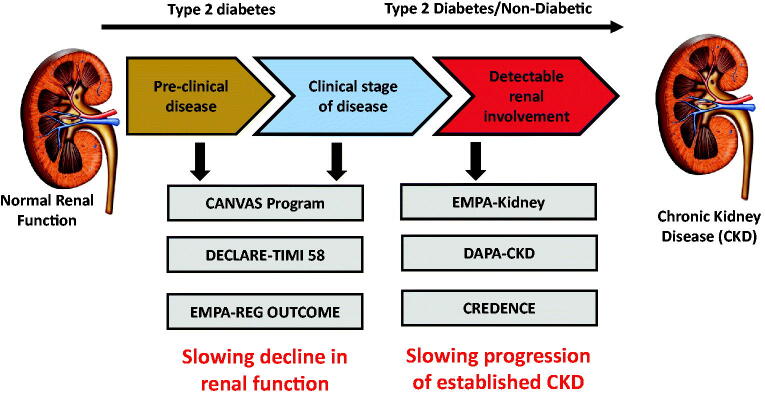
Summary of the main studies with SGLT2 inhibitors and renal outcomes.

The collective renal data and clinical trial context is summarised in Figures 5 and 6.

### Placement of SGLT2 inhibitors in the T2D treatment pathway

Recently, a consensus report by the American Diabetes Association (ADA) and the European Association for the Study of Diabetes (EASD) provided updated recommendations on the management of hyperglycaemia [74]. Incorporating the recently amassed clinical trial evidence-base of SGLT2 inhibitors on CV and renal outcomes, the treatment algorithm proposes an initial distinction of glucose-lowering medication based on clinical characteristics and the presence or absence of co-morbidities such as atherosclerotic CVD, HF or CKD. For patients with clinical CVD, an SGLT2 inhibitor or a GLP-1 receptor agonist with proven cardiovascular benefit is recommended while for those patients with CKD or HF and atherosclerotic cardiovascular disease, an SGLT2 inhibitor with proven benefit is recommended. They also recommend consideration of an SGLT2 inhibitor or GLP1-RA where there is a compelling need to minimize weight gain or promote weight loss or to minimize hypoglycaemia. This stratification of high-risk individuals to receive SGLT2 inhibitors (or GLP1-RA) is an important departure from previous guidelines.

### Development of dual SGLT inhibition

Given the increased transport capacity of SGLT1 for glucose following SGLT2 inhibition and the significant role of SGLT1 in intestinal glucose uptake, optimizing dual SGLT inhibition is an appealing strategy. As well as being identified in the human kidney and intestine, SGLT1 is expressed in the liver, heart, skeletal muscle, lung, and possibly brain, indicative of several extra-renal functions of this transporter [75]. Mechanistic studies are needed to determine the functional role of SGLT1 at these locations.

The therapeutic potential of phlorizin was limited, due to poor oral availability, and effects on gut glucose absorption that resulted in diarrhoea. Sotagliflozin is the first dual SGLT inhibitor licenced for the treatment of T1D, in combination with insulin, in Europe. Compared to selective SGLT2 inhibitors, sotagliflozin has similar efficacy in terms of glycaemic control and weight loss and despite its increased selectivity for SGLT1 is well tolerated without severe gastrointestinal side effects, most likely due to incomplete SGLT1 inhibition [76].

In a small, proof of concept randomized controlled trial, lucagliflozin, a selective and potent dual SGLT1/SGLT2 inhibitor, demonstrated significant weight loss (5.7%) versus placebo (*p* < .001) in patients with obesity, and favourable increases in incretin hormones and urinary glucose excretion (∼100 g) in patients with T2D [77]. A higher incidence of diarrhoea was reported at higher doses of lucagliflozin, possibly explained by greater SGLT1 inhibition. Evaluation of the long-term efficacy and safety of existing or developing SGLT1 and dual SGLT inhibitors is required, in particular risk of diabetic ketoacidosis and cardiovascular outcomes.

### Combination therapy

#### GLP-1 receptor agonist

The glucose-lowering effects of SGLT2 inhibitors are attenuated due to increased endogenous (hepatic) glucose production, partially explained by hyperglucagonaemia. Combined with the concept of compensatory hyperphagia with SGLT2 inhibitor therapy, a clear rationale for the co-administration of an SGLT2 inhibitor with a GLP-1 receptor agonist is provided. The aim of combination treatment would be to offset the compensatory metabolic changes and attenuate the central hyperphagic drive and ideally achieve additive, even synergistic, weight loss and metabolic changes. Two clinical trials have examined the glucose-lowering efficacy and weight change when an SGTL2 inhibitor is combined with a GLP-1 receptor agonist in T2D [78,79]. Mechanistic explanations for weight loss associated with SGLT2 inhibitors, with or without GLP1 receptor agonists have been discussed narratively [28].

#### Combination of SGLT2 and GLP-1 receptor agonist on CVD

In the EXSCEL study, open-label SGLT2 inhibitor use, in parallel with, or shortly after once-weekly exenatide, occurred in 8.7% of participants. Those on combination therapy were propensity-matched to those on placebo only and those on once-weekly exenatide only. The risk for MACE and all-cause mortality was numerically lower, and the estimated eGFR slope improved, with combination therapy compared with placebo or once-weekly exenatide only alone supporting the hypothesis that combinatorial therapy may provide benefit on cardiovascular outcomes and mortality [80].

#### DPP-4 inhibitor

DPP-4 inhibitors are less potent than GLP-1 receptor agonists with an average HbA1c reduction of −0.74% [81] and they are considered to be weight neutral. Very small mechanistic studies with dual SGLT1/2 inhibitors have demonstrated increased circulating levels of GLP-1, presumably due to delayed SGLT1-mediated intestinal glucose absorption [77,82]. Developed to inhibit the degradation of endogenous GLP-1 and other glucoregulatory peptides, the glycaemic improvement was seen with the combination of an SGLT2 inhibitor with a DPP-4 inhibitor is modest and sub-additive [83]. Although clinically relevant improvements in HbA1c are seen, it seems that DPP4-inhibitors are unable to offset the (mal)adaptive changes in hepatic glucose production and hyperglucagonaemia seen with SGTL2 inhibitors.

#### Combination of SGLT2 and DPP4-inhibitor on kidney disease

The DELIGHT study examined the albuminuria-lowering effect of dapagliflozin, with and without saxagliptin, in patients with T2D and moderate-to-severe CKD [84]. Dapagliflozin alone or in combination with saxagliptin, versus placebo, reduced the urinary albumin-creatinine ratio (by 21 and 38% respectively).

### Adverse effects

Data is continually emerging around the adverse effects and risks associated with SGLT2 inhibitors from a variety of sources including extensive clinical experience, case series, post-marketing surveillance and regulatory websites [85]. The most common adverse effect is genital mycotic infections although there also be a slightly increased risk of urinary tract infections and there have been concerns about Fournier’s gangrene, a necrotizing fasciitis of the scrotum [86]. Other signals include diabetic ketoacidosis, foot and leg amputation and bone fracture briefly discussed below.

#### Diabetic ketoacidosis (DKA)

**Figure 7.**
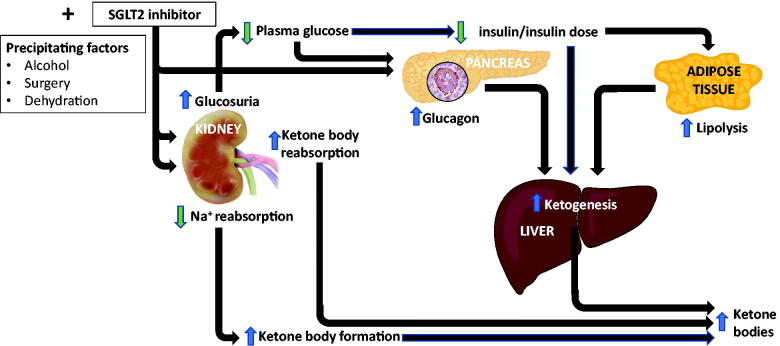
Mechanisms of diabetic ketoacidosis with SGLT2 inhibitors.

DKA, the triad of hyperglycaemia (blood glucose >11 mmol/l), acidosis (venous bicarbonate <15 mmol/l) and ketonuria/ketonaemia, represents the most common and serious hyperglycaemic emergency in patients with diabetes (Figure 7). Most commonly it occurs in auto-immune T1D but can also occur in poorly controlled, insulin-deficient patients with T2D [87]. In 2015, regulatory authorities (the Federal Drug Administration and the European Medicines Agency) first advised clinicians that the use of SGLT2 inhibitors was associated with an increased risk of diabetic ketoacidosis (DKA) (Figure 7). In one analysis from the US FDA Adverse Event Reporting Systems (FAERS), 71% presented with normal or only mildly elevated plasma glucose concentrations, euglycaemic ketoacidosis (EuDKA) [88], with relative normoglycaemia presumably maintained by augmented renal glycosuria. This is an important practical issue as minimal hyperglycaemia may result in a delay in the diagnosis in >50% of individuals with lesser degrees of dehydration given the lack of hyperglycaemia. Further analysis from FAERS subsequently reported >2500 cases of DKA in which SGLT2 inhibitors were listed as suspect or concomitant drugs [89]. Case series have helped identify common themes, and in particular precipitating events including intercurrent illness, for example, vomiting/diarrhoea, dehydration, discontinuation or reduction of insulin dosage related to glycaemic improvement, surgery, fasting or ketogenic/low carbohydrate diet and excessive alcohol [90]. A recent meta-analysis of 39 RCTs, involving >60,000 patients with T2D, confirmed SGLT2 inhibitors were statistically associated with an increased risk of DKA versus control (OR 2.13, 95% CI 1.38, 3.27) [91]. However, it is generally considered to be a rare event. In contrast, DKA, in the context of T1D is more common, with or without the use of SGLT2 inhibitors, but off-label use of SGLT2 inhibitors, especially among young females was a risk factor [92]. The rates of DKA and ketosis with SGLT2 inhibitors in T1D may be between 5–10% based on clinical trial data in T1D [93].

There are multiple biologically plausible mechanisms by which SGLT2 inhibitors cause ketosis or DKA in T1D or T2D (illustrated in Figure 7). The lowering of plasma glucose will lead to lower insulin levels, coupled with an increased glucagon secretion associated with SGLT2 inhibitors, with a consequently increased glucagon: insulin ratio. This raised glucagon: insulin concentration drives lipolysis, fatty acid oxidation and ketone production by the liver. Simultaneously, increased ketone body reabsorption may occur through the kidney secondary to renal glucose loss. All of this may be compounded by a reduction in insulin dosage or even discontinuation of insulin in patients with T1D, or in patients with T2D who are insulin deficient.

Patients with T2D and particularly those with T1D being initiated on SGLT2 inhibitors need to be carefully counselled about this potential risk, the associated symptoms and educated about ketone monitoring. Recently international consensus guidelines have been compiled to mitigate the risk of the associated DKA risk in patients with T1D being treated with SGLT2 inhibitors [94]. Any patient with T1D or T2D who experiences nausea, vomiting, is generally unwell or develops a metabolic acidosis in the setting of SGLT2 inhibitor therapy, should undergo prompt evaluation of ketonuria/ketonaemia.

##### Lower limb amputation

In CANVAS there was an increased risk of lower limb amputation observed with canagliflozin, primarily at the level of the toe or metatarsal, although this has not been reproduced in other studies with other SGLT2 inhibitors, nor was any concern noted regarding canagliflozin use in CREDENCE [66,67]. It is unclear to what extent canagliflozin increases the amputation risk, if at all, but caution is advised in patients with risk factors for lower limb amputation. Risk factors for amputation with SGLT2 inhibitors include those with a previous history of amputation or foot ulcers, peripheral vascular disease and neuropathy and those with baseline CVD.

##### Bone health

The association between SGLT2 inhibitors and the risk of fractures is based on findings from CANVAS, which found a significantly increased risk of fractures compared with placebo (HR 1.26; 95% CI 1.04,1.52) [66]. Biologically plausible mechanisms would underlie this including elevated serum phosphate levels or reduced bone mineral density. These findings were not reproduced in CREDENCE where there was a similar fracture risk in both groups [67]. In a subsequent population-based cohort study in the UK, the use of SGLT2 inhibitors was not associated with an increased risk of fractures compared with the use of DPP-4 inhibitors [95].

## Summary

SGLT2 inhibitors have become an indispensable part of the therapeutic armamentarium in T2D with useful metabolic effects in all patients with little or no risk of hypoglycaemia. Their role in T1D is emerging with a need for careful patient selection and a need for ongoing pharmacovigilance. In T2D, they have a very clear role emerging in high-risk patients with atherosclerotic CVD, HF and CKD and their use is expanding to other patient groups who do not have diabetes, particularly those with HF of chronic kidney disease. Results of further ongoing randomized controlled trials will, no doubt, expand the clinical indications for these drugs in diverse patient populations and we can continue on this exciting journey of discovery.
